# The Role of Exosomes as Mediators of Neuroinflammation in the Pathogenesis and Treatment of Alzheimer’s Disease

**DOI:** 10.3389/fnagi.2022.899944

**Published:** 2022-06-28

**Authors:** Shiting Weng, Qi-Lun Lai, Junjun Wang, Liying Zhuang, Lin Cheng, Yejia Mo, Lu Liu, Zexian Zhao, Ying Zhang, Song Qiao

**Affiliations:** ^1^The Second Clinical Medical College, Zhejiang Chinese Medicine University, Hangzhou, China; ^2^Department of Neurology, Zhejiang Hospital, Hangzhou, China; ^3^Department of Neurology, Second Affiliated Hospital of Zhejiang University, Hangzhou, China

**Keywords:** Alzheimer’s disease, neuroinflammation, exosomes, neuroglia, beta-amyloid, tau, therapeutic target

## Abstract

Alzheimer’s disease (AD) is a common neurodegenerative disease characterized by progressive dementia. Accumulation of β–amyloid peptide 1–42 and phosphorylation of tau protein in the brain are the two main pathological features of AD. However, comprehensive studies have shown that neuroinflammation also plays a crucial role in the pathogenesis of AD. Neuroinflammation is associated with neuronal death and abnormal protein aggregation and promotes the pathological process of β-amyloid peptide 1–42 and tau protein. The inflammatory components associated with AD include glial cells, complement system, cytokines and chemokines. In recent years, some researchers have focused on exosomes, a type of membrane nano vesicles. Exosomes can transport proteins, lipids, microRNAs and other signaling molecules to participate in a variety of signaling pathways for signal transmission or immune response, affecting the activity of target cells and participating in important pathophysiological processes. Therefore, exosomes play an essential role in intercellular communication and may mediate neuroinflammation to promote the development of AD. This paper reviews the occurrence and development of neuroinflammation and exosomes in AD, providing a deeper understanding of the pathogenesis of AD. Furthermore, the role of exosomes in the pathogenesis and treatment of AD is further described, demonstrating their potential as therapeutic targets for neuroinflammation and AD in the future.

## Introduction

Alzheimer’s disease (AD) is a common neurodegenerative disease in the elderly, and is one of the main causes of dementia. AD is a global problem. At present, about 24 million people are affected by the disease and it is estimated that this number will quadruple by 2050. AD is mainly caused by protein misfolding and aggregation (Tran and Ha-Duong, 2015; Tiwari et al., 2019), altering their conformation and causing gradual aggregation, eventually leading to neuronal dysfunction or even death (Ke et al., 2017). The two hallmark histopathological features of AD are plaque formation caused by amyloid-beta (Aβ) deposition and neurofibrillary tangle (NFT) formation with hyperphosphorylated tau (p-tau) (Huat et al., 2019). Recent studies have found that in addition to these two pathological features, neuroinflammation, neuron loss, aging, gene mutation, metabolism and oxidative stress could also promote the development of AD (Yin et al., 2020), especially neuroinflammation, which is considered to be the third characteristic feature of AD.

At present, more and more researchers have focused on the role of exosomes in inflammatory diseases of the central nervous system (CNS). In the complex intercellular communication system, exosomes are the smallest membranous nanovesicles originating from endosomes. Exosomes are secreted by multiple types of cells and regulate a variety of signal pathways through the transmission of various signal molecules, participating in the information exchange between cells (Valadi et al., 2007; Yin et al., 2020). There are specific molecular markers on the surface membrane of exosomes, which can be traced back to the original cells, and can potentially be used as molecular markers for the diagnosis of some diseases. In addition, exosomes can carry molecules across the blood-brain barrier (BBB). They have a stable lipid bilayer membrane structure, which makes them mobile. Furthermore, exosomes are small nano-sized molecules, which facilitate the entry through the BBB (Valadi et al., 2007). In other words, exosomes take part in cellular communication in multiple neurological diseases, participate in the pathogenesis of these diseases, including AD, and can be used as targets for diagnosis and treatment. This review systematically describes the neuroinflammation process and the role of exosomes in the pathogenesis of AD.

## The Main Pathogenesis of Alzheimer’s Disease

The accumulation of Aβ is one of the main causes of AD neurodegeneration. Aβ accumulates in the neocortex area of the orbital frontal cortex, temporal lobe and basal cortex, and gradually spreads into the whole neocortex, hippocampus, amygdala, diencephalon and basal ganglia. In severe cases, Aβ also involves the middle brain, the inferior brainstem and the cerebellum cortex. Aβ originates from the sequential cleavage of amyloid precursor protein (APP). Cleavage by β-secretase within the luminal/extracellular domain generates β-carboxyl-terminal fragments. Following β-secretase cleavage, γ-secretase processes APP at the carboxyl terminus to produce Aβ (Huat et al., 2019). These insoluble Aβ fibrils accumulate and spread to the synaptic gap, which interferes with synaptic signal conduction (Rabbito et al., 2020; Wang et al., 2020), leading to the formation of insoluble plaques. This polymerization also leads to kinase activation, resulting in hyperphosphorylation of the tau proteins which are involved in microtubule stabilization. This leads to the dissociation of microtubules and axonal transport dysfunction (Dixit et al., 2008; Zhang et al., 2021), which makes tau protein gradually gather to form NFT, causing abnormal communication and signal conduction of neurons, and eventually apoptosis of neurons (Sung et al., 2020; Dregni et al., 2022). After the formation of plaques and fiber tangles, microglia and astrocytes gather around the plaque, promote the activation of glial cells and local inflammatory reactions, and contribute to neurotoxicity (Tiwari et al., 2019). The severity of these two pathological features is positively correlated with the degree of dementia degree in AD.

In addition to Aβ and NFT, neuroinflammation is the third core neuropathological feature of AD (Heneka et al., 2015; Calsolaro and Edison, 2016; Piirainen et al., 2017; Aminzadeh et al., 2018). Neuroinflammation responds to neuronal loss or abnormal protein aggregation. Many studies have reported persistent neuroinflammation in the early stage of AD, which promotes the formation of Aβ and NFT and the toxicity and death of neurons (Garwood et al., 2011; Piccioni et al., 2021). A large number of studies have demonstrated chronic inflammation of the CNS in AD (Rubio-Perez and Morillas-Ruiz, 2012; Sarlus and Heneka, 2017). Activated glial cells, especially microglia and astrocytes, play a central role in the pathogenesis of AD. They are usually found near neurons and plaques (Sarlus and Heneka, 2017), and can cause the release of inflammatory factors and cytotoxins, including cytokines, chemokines and complement factors (Rubio-Perez and Morillas-Ruiz, 2012; Sarlus and Heneka, 2017). As mentioned earlier, this inflammatory response can be caused by the accumulation of Aβ and pathological tau protein formation.

## Neuroinflammation in Alzheimer’s Disease

Inflammation represents a response induced by injury or destruction of tissues, which enables removal, dilution, or isolation of both injurious substances and injured tissue. Inflammation can be classified as either acute or chronic. As a common inflammatory process, acute neuroinflammation occurs immediately following injury to the CNS (Cai Z. Y. et al., 2018). It is characterized by the release of inflammatory molecules, glial cell activation, endothelial cell activation tissue edema and so on (Fullerton and Gilroy, 2016; Laurent et al., 2018). Chronic neuroinflammation is of longer duration, with maintained glial cell activation and recruitment of other immune cells in the brain. More and more evidences have suggested that AD is associated with chronic inflammatory responses, with sustained presence of inflammatory cytokines from activated microglia and astrocytes, free radicals, and oxidative stress (Kaur et al., 2019; Ozben and Ozben, 2019; Poudel and Park, 2022).

### Microglia

Microglia show both beneficial and harmful effects in AD. On the one hand, activated microglia reduce the deposition of Aβ by phagocytosis (Frautschy et al., 1998; Yamamoto et al., 2018), secrete neurotrophic factors, and promote the survival of neurons and tissue repair (Gehrmann et al., 1995; Liu and Hong, 2003). On the other hand, the increase of local cytokine concentration in AD patients causes the downregulation of Aβ phagocytic receptor expression in microglia, resulting in the accumulation of Aβ and the decrease of Aβ clearance rate. Furthermore, the toll-like receptor (TLR) on microglia is activated, resulting in the activation of microglia and the secretion of pro-inflammatory cytokines and chemokines (Heneka et al., 2015). This over-activation usually occurs under chronic conditions, which not only produces pro-inflammatory mediators and cytotoxicity but also leads to the prolongation of neuroinflammation. The continuous formation of Aβ, inflammation, and activated microglia form a positive feedback circuit, aggravating the severity of AD (Hickman et al., 2008; Hansen et al., 2018; Prinz et al., 2019). In addition, reactive microglia gather around the cells forming NFT, indicating that tau protein phosphorylation is also related to inflammatory response (Azevedo et al., 2013). Multiple studies have shown that chronic levels of inflammatory mediators aggravate the activation of key protein kinases that control tau phosphorylation (Azevedo et al., 2013; Ahmad et al., 2022). For example, long-term release of tumor necrosis factor-α (TNF-α) from microglia has been shown to induce tau aggregation in neurons *in vitro* (Azevedo et al., 2013; Heneka et al., 2015; Ahmad et al., 2022).

### Astrocytes

Astrocytes are multi-functional cells involved in the nutrition of nerves, waste removal, signal transmission and maintenance of BBB homeostasis (Newcombe et al., 2018; Sung et al., 2020). Reactive astrocyte proliferation and atrophy are the pathological characteristics of astrocytes, which occur in the early stage of AD, even before Aβ deposition (Fleeman and Proctor, 2021). Glial fibrillary acidic protein usually elevated in AD, indicating astrocyte activation (Olabarria et al., 2011; Ahmad et al., 2022). Similar to microglia, activated astrocytes also release cytokines, interleukin (IL), nitric oxide (NO) and other cytotoxic molecules, which exacerbate the process of neuroinflammation (Lian et al., 2016). It is worth mentioning that astrocytes, capillary endothelial cells and perivascular cells all participate in the formation of the BBB. Activated astrocytes enhance BBB permeability by promoting inflammation and disrupting its normal physiological function, resulting in the imbalance of Aβ clearance at the BBB from the brain parenchyma into the blood (Erdő et al., 2017), and promoting Tau pathology and neuroinflammation (Kumfu et al., 2018). At the same time, astrocytes play a key role in the transport of Aβ through BBB, which is regulated by receptor for advanced glycation endproducts (RAGE) and low-density lipoprotein receptor-related protein 1 (LRP-1) in endothelial cells (Patterson et al., 2018). Astrocyte dysfunction seems to promote RAGE (transport of Aβ into brain across the BBB) activity and decrease LRP-1 activity (brain-derived Aβ enters the bloodstream *via* BBB) (Askarova et al., 2011).

### Cell Factor

It can be said that microglia and astrocytes are the main sources of cytokines in AD. Many studies have shown that the increase of Aβ deposits and tau protein phosphorylation is related to the increase of pro-inflammatory cytokines (Heneka et al., 2015; Sung et al., 2020). These pro-inflammatory cytokines include IL-1α, IL-1β, IL-6, interferon- α (IFN-α), TNF-α and granulocyte-macrophage colony-stimulating factor (GM-CSF) (Rubio-Perez and Morillas-Ruiz, 2012; Babcock et al., 2015). Among them, IL-1α and IL-1β can regulate the synthesis and secretion of APP, promote the production of Aβ (Akama and Van Eldik, 2000), and increase the phosphorylation of tau protein through the MAPK-p38 pathway (Han et al., 2017; Khezri et al., 2022). IL-6 stimulates astrocyte proliferation (Lee et al., 2021), activates microglia (West et al., 2022), increases APP expression (Tsatsanis et al., 2021), and increases tau phosphorylation through the cdk5/p35 pathway (Huang et al., 2022). TNF-α is secreted by activated microglia and can damage nerve cells by enhancing NMDA receptor-mediated neurotoxicity (Zou and Crews, 2005). GM-CSF can be induced by TNF-α to enhance the inflammatory response (Hansen et al., 2018), while IFN-α can increase the activity of TNF and recruit NO to induce inflammation (Rubio-Perez and Morillas-Ruiz, 2012).

### Other Pro-inflammatory Factors

In AD, chemotaxis might be responsible for attracting glial cells toward the neuritic plaque and inducing inflammation in this region (Savarin-Vuaillat and Ransohoff, 2007). Aβ reportedly activates astrocytes and oligodendrocytes to produce chemokines, in particular monocyte chemotactic protein-1 and RANTES (CCL5), which serve as potent *in vitro* microglial and macrophage chemoattractants (Lee et al., 2010; Paolicelli et al., 2011; Vandendriessche et al., 2021). In addition to chemokines, complement factors are also involved in the inflammatory process. Complement factor activation leads to inflammatory stimulation. C3a, C4a, and C5a combines with the receptor on the microglia membrane, causing a respiratory burst and producing a large number of oxygen free radicals, which damage neurons (Bonifati and Kishore, 2007). These inflammatory reactions accelerate the formation of senile plaques and eventually develop into AD (Bonifati and Kishore, 2007).

## Overview of Exosomes

Exosomes are lipid bilayer vesicles with a diameter of 30–150 nm, which can carry specific proteins, lipids, mRNA, miRNA and other substances. Exosomes can be secreted by most cells of the body, including B cells, T cells, dendritic cells, macrophages, neurons, glial cells, most tumor cell lines and stem cells, etc. They are naturally found in body fluids, including blood, saliva, urine, cerebrospinal fluid, and breast milk (Abels and Breakefield, 2016; Pascual et al., 2020).

The origin, synthesis and secretion of exosomes go through the following processes. The protocell membrane forms early endosomes by endocytosis or “budding inward,” which then gradually mature inside the cell into late endosomes and multivesicular bodies (MVBs). MVB content can be transported to the lysosome complex where it is degraded or be reserved as temporary storage inside the cell or translocated to the plasma membrane. The MVBs then fuse with the cell membrane and exit the cell as exosomes (Colombo et al., 2014).

**FIGURE 1:**
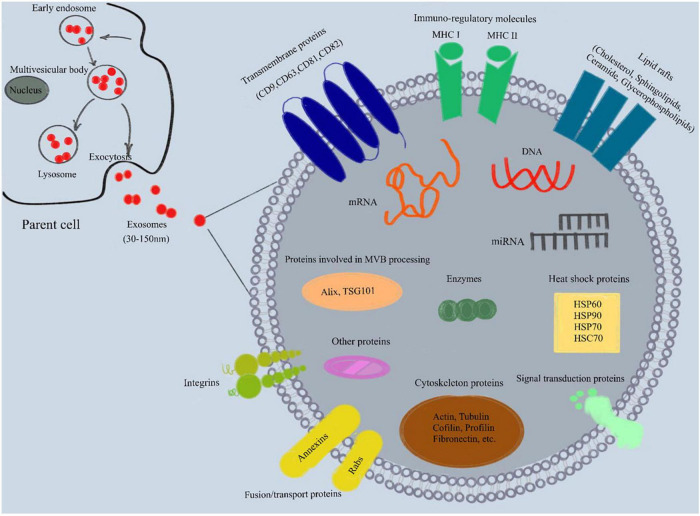
Composition of exosomes. Exosomes are lipid bilayer vesicles with a diameter of 30–150 nm, which can carry specific proteins, lipids, mRNA, miRNA and other substances. In addition, exosome membrane is rich in lipid rafts (cholesterol, sphingolipids, ceramide and glycerophospholipids). Exosome proteins include four transmembrane proteins (CD9, CD63, CD81, CD82), heat shock proteins (HSC70, HSP60, Hsp70, Hsp90), proteins involved in MVB processing (Alix, TSG101), cytoskeleton proteins (actin, tubulin, cofilin, profilin, fibronectin, etc.), fusion/transport proteins (Annexins, Rabs), integrins, signal transduction proteins, immune regulatory molecules (MHC I and II) and various metabolic enzymes. MHC, major histocompatibility complex; mRNA, messenger RNA; miRNA, microRNA; MVB, multivesicular body.

Exosome membranes are mainly composed of phospholipids and proteins. The membrane is rich in lipid rafts, including cholesterol, sphingolipids, ceramide and glycerophospholipids (Ohno et al., 2013; Raposo and Stoorvogel, 2013). Exosome proteins include four transmembrane proteins (CD9, CD63, CD81, CD82), heat shock proteins (HSC70, HSP60, Hsp70, Hsp90), proteins involved in MVB processing (Alix, TSG101), cytoskeleton proteins (actin, tubulin, cofilin, profilin, fibronectin, etc.), fusion/transport proteins (Annexins, Rabs), integrins, signal transduction proteins, immune regulatory molecules (MHC I and II) and various metabolic enzymes (Figure 1; Koppers-Lalic et al., 2013; Kalani et al., 2014; Wu et al., 2017). Meanwhile, exosomes also carry a variety of nucleic acids (mainly RNA, such as mRNA, miRNA, piRNA, snoRNA, snRNA, rRNA, tRNA, Y-RNA, scRNA, etc.) and DNA (Koppers-Lalic et al., 2013; Raposo and Stoorvogel, 2013).

## The Physiological Function of Exosomes in the Central Nervous System

**FIGURE 2:**
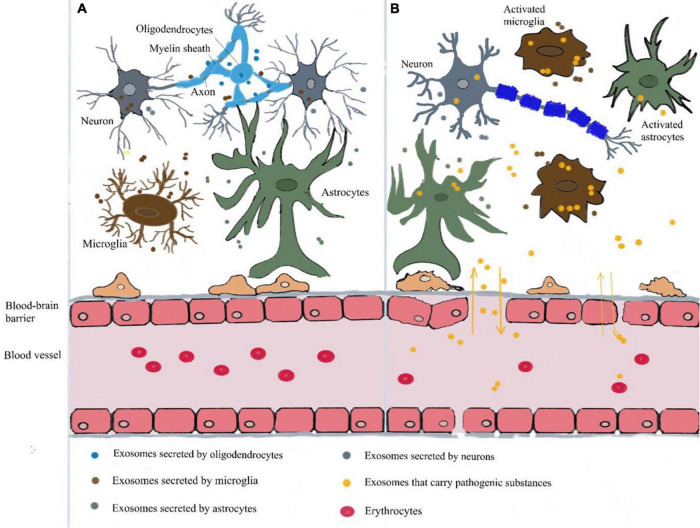
Roles of exosomes in the healthy brain and a neuroinflammatory state of AD. **(A)** Healthy brain. Exosomes mediate normal intercellular communication in the brain. Glial-derived exosomes mediate important functions participating in neural circuit development and maintenance, promoting neurite outgrowth, synaptic activity and neuronal survival. Oligodendrocytes-derived exosomes provide trophic support to axons facilitating myelination. **(B)** Neuroinflammatory state of AD. In Alzheimer’s disease (AD), as an inflammatory mediator, exosomes induce neuroinflammation through information exchange between neurons and glial cells. They can diffuse in interconnected neurons and transport amyloid-beta (Aβ) and tau proteins through the endosomatic pathway and axonal transport. At the same time, exosomes can cause neurological dysfunction by carrying pathogenic substances such as malregulated miRNA, mRNA and proteins. These exosomes are able to cross the blood-brain barrier propagating the neuroinflammatory response to the periphery. Similarly, plasma exosomes can also enter the brain and target neurons and glial cells, causing a series of pathophysiological reactions.

In the CNS, glial cells, stem cells and neuron cells can secrete exosomes (van Niel et al., 2006; Younas et al., 2022). Exosomes secreted by these cells under normal or pathological conditions can be isolated from human brain tissue and cerebrospinal fluid (Cai et al., 2017; Verheul et al., 2017). Exosomes secreted by nerve cells also transmit signals to other nerve cells and impact the development of the CNS, regulation of synaptic activity and regeneration of nerve injury. For example, neurons regulate the differentiation of oligodendrocytes by affecting the release of exosomes from oligodendrocytes, providing nutrition for axons and promoting myelin formation (Frühbeis et al., 2013). Glial cells secrete synaptophysin related to nerve development under stress conditions (Wang et al., 2011). In addition, microglia-derived exosomes can also increase the metabolism of ceramide and sphingosine in receptor neurons, resulting in the increase of neurotransmitters (Podbielska et al., 2016). In AD, exosomes can stimulate the phagocytosis of microglia and participate in the process of neuronal remodeling (Pascual et al., 2020; Figure 2).

## The Role of Exosomes in Neuroinflammation of Alzheimer’s Disease

As mentioned above, exosomes are involved in neuroinflammation, which triggers beta-amyloid pathogenesis and tau hyperphosphorylation (Ridder et al., 2014). Exosomes can carry Aβ, tau, prions, and α-synuclein, and can spread pathogenic proteins across the brain (Saeedi et al., 2019; Aheget et al., 2020; Figure 2). Furthermore, it has been shown that exosomes are strongly associated with beta-amyloid clearance (Eren et al., 2022). As an inflammatory mediator, exosomes induce neuroinflammation through information exchange between neurons and glial cells. They can diffuse in interconnected neurons and transport Aβ and tau proteins through the endosomatic pathway and axonal transport (Polanco et al., 2018). A study has found that exosomes promote Aβ aggregation and accelerate amyloid plaque formation. Meanwhile, *in vivo* exosome reduction resulted in lower amyloid plaque load in the 5xFAD mouse model, a mouse line that expresses five mutations of familial AD (Cai Z. Y. et al., 2018). On the other hand, under normal circumstances, Aβ is transported by exosomes and degraded by lysosomes, which may lead to their accumulation in exosomes and diffusion in AD (Yuyama et al., 2012; An et al., 2013). Similarly, this lysosomal dysfunction has been observed with exosomal α-synuclein release and transmission (Alvarez-Erviti et al., 2011a). Exosomes can not only spread AD pathological proteins; they are also suggested to play a harmful role in impairing neuronal functions by other means in AD. Amyloid peptides could activate neutral sphingomyelinase 2 (nSMase2) and induce an increase in the secretion of ceramide-containing exosomes in astrocytes. In contrast, these secreted exosomes could be captured by astrocytes and subsequently cause neural apoptosis. GW4869, an inhibitor of nSMase2, was shown to reduce Aβ in a mouse model of AD by preventing the secretion of exosomes, thus indicating that the ceramide generated by nSMase2 may be critical for the formation of exosomes (Wang et al., 2012).

Tau is a core protein associated with the pathogenesis of AD and is secreted in exosomes. It is reported that exosomal derived hyperphosphorylated tau concentrations are significantly increased in the late stage of AD compared to the early stage, indicating that exosomal tau may contribute to abnormal tau phosphorylation (Saman et al., 2012). In addition, studies on tau proteins reported that exosomes rich in phosphorylated tau proteins were collected from the cerebrospinal fluid of AD patients, which can promote the aggregation of tau protein in microglia and neurons (Wang et al., 2017). A clinical study showed that the exosome levels of total tau (pT181-tau and pS396-tau) were significantly higher in AD patients than in controls, suggesting that pS396-tau and pT181-tau levels in extracts of neutrally derived blood exosomes predict AD development before its clinical onset (Fiandaca et al., 2015). Another study showed that microglial cells play a significant role in phagocytosis and the secretion of tau in exosomes. The depletion of microglia in two diverse tauopathy mouse models showed that the propagation of tau could be inhibited, and that the inhibition of exosome synthesis reduced the propagation of tau compared with a control group, both *in vitro* and *in vivo*. Based on these results, exosomes derived from microglia are efficient carriers for spreading tau between neurons (Yin et al., 2020). Moreover, studies have also shown that cell lines with similar tau protein levels have been found in the postmortem brain of AD patients. Exosomes containing pro-apoptotic protein and tau protein transfer these proteins to receptor cells through astrocytes to induce nerve cell death and neurodegeneration (Reilly et al., 2017). As mentioned earlier, the accumulation of Aβ and the hyperphosphorylation of tau protein can continuously activate microglia and astrocytes, promoting the inflammatory response. The activated glial cells release exosomes, which release Aβ and tau proteins into the extracellular environment, inducing the inflammatory cascade reaction, thus enhancing the progress of inflammation.

It is worth mentioning that exosome-mediated miRNAs may be involved in AD (Bellingham et al., 2012). In the AD brain, extracellular Aβ plaques, which ultimately lead to progressive loss of neurons, are derived from the processing of APP by BACE. Significantly dysregulated miRNAs such as miR-193b, miR-101, or BACE1 like miR-29c target APP to influence Aβ generation in AD brain (Bryniarski et al., 2015). It is conjectured that miRNAs mediated by exosomes may initiate TLR activation under certain circumstances. The relationship between miRNA mediated by exosomes and TLRs was deemed important in discovering the role of exosomal miRNAs in the neuroinflammation of AD (Bryniarski et al., 2015). Furthermore, in AD mouse and human brain, miR-146a localized to the hippocampal regions is full of proinflammatory cytokines in response to TLRs. These levels constitute disease severity and suggest the link between miR-146a and inflammation-induced neuropathology (Lukiw et al., 2011).

## The Role of Exosomes in the Treatment of Alzheimer’s Disease

One of the major obstacles to the treatment of neuroinflammatory diseases is the lack of effective vectors to transport drugs or genes across the BBB. Exosomes have low immunity, congenital stability, high transport efficiency and can cross the BBB. Therefore, they can be applied as drug delivery carriers and genetic components for the treatment of neurological diseases (Lässer, 2015).

### About Mesenchymal Stem Cell—Derived Exosomes

Previous studies have shown that mesenchymal stem cell (MSC) is involved in neurogenesis, oligodendrocyte formation and axonal connection. MSC can transport substances across the BBB, transport substances to the site of nerve injury, promote nerve regeneration (Ding et al., 2018), nerve repair (Zilka et al., 2011), decrease Aβ deposition and tau-related cell death (Yun et al., 2013), and downregulate pro-inflammatory cytokines. After a series of in-depth studies, it was found that MSCs may play a therapeutic role through exosomes (Hu et al., 2015; Zhang et al., 2015; Zhu et al., 2017). Hao et al. (2014) cultured damaged cortical neurons with human adipose-derived mesenchymal stem cells (ADMSC) and showed that the conditioned medium rich in exosomes could achieve a neuroprotective effect by inhibiting neuronal apoptosis and promoting nerve regeneration (Katsuda et al., 2013; Ding et al., 2018). In this way, the CNS can be regenerated and repaired, and can limit glutamate excitotoxicity. Similarly, exosomes were extracted from the conditioned medium of mesenchymal stromal cells and injected into the rat model and it was found that exosomes reduced the damage to neurons (Xin et al., 2013). In another study, it was shown that MSC-derived exosomes reduced glial cell activation and reduced Aβ accumulation, thus improving the learning and memory function of APP/PS1 transgenic mice (Ahmed et al., 2016; Cui et al., 2016, 2018). Some reports focused on the role of neprilysin in AD pathology, supporting a scenario in which neprilysin-loaded exosomes contribute to Aβ clearance in the brain. Accordingly, a recent study demonstrated for the first time that adipose tissue-derived MSCs produce neprilysin-bound exosomes. Co-culture experiments indicated that MSC-derived exosomes contribute to lower Aβ levels secreted in N2a cells, suggesting the therapeutic potential of microvesicle-bound neprilysin for AD treatment (Nigro et al., 2016). Exosomes are secreted from human cells. The exosomes obtained from MSC culture can be used for treatment and reduce the level of cellular immunogenicity (Vakhshiteh et al., 2019). Recent studies have shown that MSC-derived exosomes have the ability to aggregate to specific neuropathological regions (Liu et al., 2015; Perets et al., 2019), which provides a basis as therapeutic agents in AD.

### Exosomes as Containers

In addition to MSC-derived exosomes that protect nerves and mitigate pathogenic proteins, we found that exosomes can also serve as containers for therapeutic substances. First, a large number of studies have shown that exosomes can transport specific proteins to alleviate the injury of nerve and the development of AD (Hara et al., 2002; Inoki et al., 2002; Zou et al., 2018). As mentioned above, nSMase2 inhibitors such as GW4869 can block exosome secretion to reduce the accumulation of Aβ and the transmission of tau protein. And recent study showed that the up-regulation of the mammalian target of rapamycin (mTOR) facilitates the release of tau into the extracellular space in an exosome-independent manner in SH-SY5Y cells (Zou et al., 2018). The mTOR complex 1 (mTORC1) also regulates the release of exosomes through a Rab27A-dependent mechanism. mTORC1 activation inhibits exosome release, while the inhibition of mTORC1 induces the release of exosomes without significantly changing cargo content, thus indicating that mTORC1 controls the release of exosomes, but not formation (Kogure et al., 2011; Bukong et al., 2014; Zou et al., 2018). Furthermore, intracerebrally administered exosomes act as potent Aβ scavengers by binding to Aβ through enriched glycans on glycosphingolipids on the exosome surface, suggesting the role of exosomes in Aβ clearance in the CNS (Yuyama et al., 2014). Moreover, the BBB is involved in the pathogenesis of AD. BBB dysfunction induces the failure of Aβ transport from the brain to the peripheral circulation across the BBB. Especially, decreased levels of LRP-1 and increased levels of RAGE at the BBB can cause the failure of Aβ transport (Askarova et al., 2011; Patterson et al., 2018). In normal human plasma, the soluble form of LRP1 (sLRP1) is the major endogenous brain Aβ “precipitate,” representing approximately 70–90% of plasma Aβ peptide. In AD, the levels of sLRP1 and its capacity to bind Aβ are reduced, which increases the free Aβ fraction in plasma. In a mouse model of AD, restoring plasma sLRP1 with recombinant LRP-IV cluster reduces brain Aβ burden and improves functional changes in cerebral blood flow and behavioral responses without causing neuroinflammation (Cai Z. et al., 2018). Therefore, using exosomes to carry plasma sLRP1 may be a new method to regulate BBB function and treat AD.

Secondly, exosomes are involved in RNA transport, and nucleic acid fragments such as miRNA and siRNA may be used to treat AD. As a special cellular vehicle, exosomes loaded with specific miRNAs may benefit from neuroplasticity under adverse environmental conditions. It was found that exosomes from MSCs transferred Mir-133b to astrocytes and neurons, which subsequently increased axonal plasticity (Xin et al., 2012). Research by Pusic and Kraig (2014) demonstrated that environmental enrichment with serum-derived exosomes containing miR-219 is critical for the production of myelinated oligodendrocytes, which can be achieved by reducing the expression of inhibitory differentiation regulators. The role of exosomes in regulating neural regeneration enhances the recovery of learning and memory in AD patients. SiRNA is a small non-coding RNA sequence that inhibits gene expression by degrading complementary mRNA transcripts. Alvarez-Erviti et al. (2011b) demonstrated that exosomes secreted by dendritic cells inhibit target genes in the brain by delivering siRNA to neurons, microglia and oligodendrocytes. Some studies have found that exogenous siRNA transferred into the exosomes of AD mice resulted in abnormal protein expression, while the deposition of Aβ in mouse brain was significantly reduced (Alvarez-Erviti et al., 2011b). Another study showed that miR-219 directly binds to the 3’-UTR of tau mRNA and inhibits tau synthesis (Chen et al., 2017). This provides evidence for the efficacy of siRNA and miRNA in the treatment of this neurodegenerative disease.

### Interaction Between Exosomes and Microglia

Recently, more and more studies have focused on the enrichment of plasma exosomes into microglia (Fitzner et al., 2011; Ginini et al., 2022; Loch-Neckel et al., 2022). Microglia, resident immune cells in the brain, engulf dead cells and help clear out misfolded aggregates of proteins, such as amyloid plaques in AD. Plasma exosomes injected into 17-month-old AD mice were observed to aggregate around Aβ plaques and preferentially targeted microglia (Fitzner et al., 2011). Extracellular Aβ plaques are usually surrounded by activated microglia. More interestingly, most exosomes clustered around Aβ plaques were located in activated microglia, suggesting that microglia may prevent the proliferation of exosome-bound disease-causing proteins to other cells by phagocytosing. Another study found that curcumin-loaded exosomes could be rapidly transported to rat brain by intranasal administration, and induce apoptosis of activated microglia, thus delaying LPS-induced brain inflammation in mice (Zhuang et al., 2011). This provides a new therapeutic idea for alleviating neuroinflammation.

Progress in exosome research has deepened our understanding, but there are still many challenges to be solved in order to apply exosomes in clinical practice. For example, the specificity of exosome targeted delivery, the administration site, the administration frequency, the bioavailability and half-life of exosomes and the potential toxicity to non-target sites should be further studied.

## Conclusion

Growing evidence shows that neuroinflammation plays an important role in the pathology of AD. Recent studies have demonstrated that continuously activated microglia and astrocytes promote the progress of neuroinflammation and stimulate the release of various pro-inflammatory factors. The paracrine and autocrine signal transduction of pro-inflammatory factors such as cytokines also stimulate glial cells, prolonging neuroinflammation. Exosomes have been proved to be an important substance in the pathogenesis of AD as a mediator of neuroinflammation. Exosomes play an essential role in the occurrence, development, diagnosis and treatment of AD. This review summarizes the intercellular communication processes in which exosomes carry genetic material and misfolded proteins, and proposes the potential of exosomes as therapeutic agents for AD. Further evidence is required to prove the positive role of exosomes in neuroinflammation and treatment of AD and provide a safe and effective method for AD targeted therapy.

## Author Contributions

SW and Q-LL equally contributed to the study design of this review. SW, Q-LL, and SQ equally performed the literature search and wrote the manuscript. JW, LZ, LC, YM, LL, ZZ, and YZ profoundly enriched the manuscript by adding important intellectual content. All authors contributed to the article and approved the submitted version.

## Conflict of Interest

The authors declare that the research was conducted in the absence of any commercial or financial relationships that could be construed as a potential conflict of interest.

## Publisher’s Note

All claims expressed in this article are solely those of the authors and do not necessarily represent those of their affiliated organizations, or those of the publisher, the editors and the reviewers. Any product that may be evaluated in this article, or claim that may be made by its manufacturer, is not guaranteed or endorsed by the publisher.
